# Is there a problem with quantum models of psychological measurements?

**DOI:** 10.1371/journal.pone.0187733

**Published:** 2017-11-08

**Authors:** Jerome Busemeyer, Zheng Wang

**Affiliations:** 1 Psychological and Brain Sciences/Indiana University, Bloomington, Indiana, United States of America; 2 School of Communication, Transitional Data Analytics Institute /The Ohio State University, Columbus, Ohio, United States of America; Southwest University, CHINA

## Abstract

This article presents the results of an experiment, called the ABA experiment, designed to test a fundamental prediction of quantum probability theory when applied to human judgments and decisions. The prediction concerns the effect of one measurement on another when the measurements are incompatible (i.e., the answers to the measurements depend on the order of these measurements). After an initial measurement of an opinion on an issue, A, the answer to a second measurement on the same issue A immediately afterwards will certainly be the same as the first. However, according to the uncertainty principle, if a measurement of opinion on issue A is followed by an incompatible measurement on another issue, B, then the answer to a second measurement on issue A will become uncertain. This prediction was tested with 325 participants on a wide range of 12 different set of issues that were previously shown to be incompatible. Contrary to previous claims published in this journal, the empirical findings support the prediction of quantum probability theory applied to human judgments.

## Introduction

Quantum (Von Neumann) probability theory is an axiomatic theory of probability, but its axioms differ from those of classical (Kolmogorov) probability theory [1]. Although quantum theory was initially developed for particle physics, the abstract probability theory formed out of that development is completely general, and can be applied to fields outside of physics including psychological [2] and social [3] sciences. While the detailed implementations may be different, these psycho-social applications of quantum probability theory still rely on the same abstract mathematical principles as used in physics.

One important principle is the *projection postulate* of quantum theory. When applied to human judgments, it is described as follows: Suppose that prior to a measurement of an issue A, the decision maker is uncertain (i.e., superposed) with respect to the appropriate answer for this issue; after the making and reporting a decision, this uncertainty is resolved so that an immediate repetition of the measurement on issue A would certainly generate the same answer again. Formally, upon measurement of an answer to issue A, the quantum state representing the decision maker’s opinion is projected onto the subspace representing the answer that was chosen (and re-normalized). A second important principle concerns the *compatibility* of measurements. Compatibility is a technical property in quantum theory (not to be confused with its natural language interpretation): If the measurement of two issues, A and B, does not depend on their order, then the measurements are compatible; but if they are order dependent, then they are incompatible. Formally, if the projectors representing measurements A, B do not commute, then the measurements are incompatible. A third basic principle, which follows from the *uncertainty principle*, is that if the measurement of A is followed by an incompatible measurement of B, then the answer to a second measurement of A is no longer guaranteed to be the same as the first answer to issue A. The incompatible measurement of B can disturb the projection produced by the first measurement, and introduce dispersion back into the state with respect to issue A.

Recently, the conjunction of these principles has been called into question when applied to psycho-social measurements [4]. The argument is actually an empirical prediction about the results of a hypothetical experiment, called the ABA experiment. Suppose that the incompatibility of issues A, B is established in an initial experiment by testing for order effects. Then another experiment is conducted: A is tested, followed by B, and then A is retested. The quantum model must predict that the correlation *r*_*B*_(*A*_1_, *A*_2_) between the two replicates must be less than one. The authors in [4] claim that this prediction is false for many psychological judgments, and instead people would remain consistent across replications, so that the correlation is perfect. However, they did not empirically test this prediction, and as far as we know, there are no experiments that directly test these competing predictions. The purpose of this article is to report an empirical study designed to test the quantum predictions for the ABA experiment.

There are a couple of challenges that we need to overcome when conducting the ABA experiment testing. First of all, the prediction of the quantum model is contingent on the assumption that the decision maker actually makes a real second judgment about A. Formally, given the state after answering question B, a third projection of the state on to an answer for question A is made. Contrary to this assumption, it is of course possible that participants simply recall and repeat the first answer without making the effort to make a second judgment. This could occur because the participants desire to appear consistent, or because they simply wish to quickly finish the experimental questioning. Therefore, we need to use methods in the experiment to encourage making a second judgment at the moment rather than simply recalling an earlier answer.

A second difficulty arises from the description of the predictions for the quantum model. Suppose the results of the ABA experiment reveal a correlation less than but close to one. Which prediction would such a result support? How much variability in answers does a quantum model predict? One way to address this might be to compare the ABA condition to a control condition (called an AA condition) that simply repeats questions about issue A back to back. Quantum theory predicts a higher repeat rate for the AA condition as compared to the ABA condition. However, this is not a good method because it fails to keep the timing between A questions constant. Furthermore, the AA condition is more vulnerable to the problem of simply recalling and repeating an earlier answer, as mentioned above. Instead, a better way to address this problem is to vary the “incompatibility” of issue B. Formally, compatibility is an all-or-none property. In practice, however, some measurements can produce more disturbance than others. Therefore, we used a variety of pairs of measurements that were designed to produce various amounts of interference. Importantly, to address the question about how much variability a quantum model predicts, we used a previously established quantum model for the ABA experiment to derive a qualitative prediction relating the correlation between the A, B measurements to the correlation between the A, A measurements.

## A quantum model for the ABA experiment

In our earlier work [5], we developed and empirically tested a quantum model for order effects that used rating scale measurements similar to those used the present experiment. This previously established model is now used to provide new predictions for the present experiment. This section briefly describes the basic assumptions of the model. See S1 Appendix for details used to construct the unitary matrices, and see http://mypage.iu.edu/~jbusemey/quantum/HilbertSpaceModelPrograms.htm for Matlab programs.

The quantum model used here was originally designed for 9-point rating scales. Although 9-point rating scales are commonly used, the number of scale values is somewhat arbitrary, and other scales can be used, such as a coarse binary scale, or a more refined 20-point scale. A person is capable of using any of these scales, and his or her state of belief doesn’t depend on the arbitrary choice of scale selected to assess these beliefs. So a state representation is required that applies to them all. Therefore, we assume that a person is capable of evaluating the statements on a fine internal scale comprised of *N* evaluation states, ranging from state 1 (the lowest belief state) and increasing by increments of one unit up to state *N* (the highest belief state). The first *n*_1_ evaluation states are assigned the first observed rating score equal to *x* = 1, then next *n*_2_ states are assigned the next observed rating score equal to *x* = 2, and so on. For a 9-point rating scale, the last *n*_9_ states are assigned the observed rating score equal to *x* = 9. In the previous model, it was assumed that judges are capable of using a very fine equally spaced lattice. More specifically, we set states (1, 2⋯, 11) to rating *x* = 1, and (12, 13⋯, 22) to rating *x* = 2, and so on up to (88, 89⋯, 99) to rating *x* = 9, which produces a total of *N* = 9 ⋅ 11 = 99 evaluation states. We used an odd number for each category to allow for a midpoint within each category. We chose *N* = 9 ⋅ 11 = 99 states because it approximates a continuum, and increasing the number *n*_*k*_ of states assigned to each rating produces practically the same results.

Therefore, the quantum state of the decision maker is represented by a *N* = 99 dimensional unit length belief state vector, *ψ*. Each coordinate of *ψ* represents a degree of belief in a statement with beliefs increasing from a minimum to a maximum degree across the coordinate indices. The amplitude assigned to a coordinate represents the potential to select a particular degree of belief.

Intuitively, the quantum model operates like an “anchoring and adjustment” procedure (e.g., [6]): The answer to a question provides an anchor that is then adjusted in light of a subsequent question. In other words, the anchor sets up a context that then carries over and influences the second question.

Before a statement about an issue is presented, the judge is assumed to be in a state that of no opinion with respect to each issue, symbolized by the vector *ψ*_0_. Ratings based on this initial state are distributed evenly around the middle of the neutral point of the rating scale.

A statement provides information for evaluating an issue, and this evaluation process is represented by a unitary operator, symbolized as *U*_*A*_, that “rotates” up or down the belief states depending on the direction and strength of the perceived validity of the statement. The state following the evaluation of statement A equals $U_{A}^{†}\cdot \psi_{0}$. This is the state before the first measurement of A occurs.

The rotated state following the evaluation of statement A determines the probabilities of answers to question A. The probability of selecting a value *x* on the rating scale is obtained by (1) selecting the coordinates of the state that are mapped onto the rating value *x*, which is called projection, and then (2) squaring the magnitude of the projection. Formally, if we define *M*_*x*_ as the projector that picks the coordinates for *x*, then probability of selecting the rating value *x* equals $p\left(A_{1}=x\right)={\left\|M_{x}\cdot U_{A}^{†}\cdot \psi_{0}\right\|}^{2}$.

After selecting the first rating response to statement A, the belief state is revised and consistent with this first response, which provides the anchor for the anchoring—adjustment process. This is the part of the measurement process that uses the projection postulate. Formally, if *x* is chosen, the state is updated to $\psi_{x}=\frac{M_{x}\cdot U_{A}^{†}\cdot \psi_{0}}{\sqrt{p(A_{1}=x)}}$. If question A was measured a second time at this point (before question B), then the probability of getting the same answer is 1.0.

To evaluate the second question, B, the anchor provided by the previous state *ψ*_*x*_ undergoes an adjustment process, represented by the unitary operator *U*_*BA*_ ⋅ *ψ*_*x*_, which evolves the state to another state reflecting the perceived validity of statement B. The state after the adjustment to B is then used to select a rating for the question about B.

The state after answering B is now changed from the state after the answer to A the first time. This is where the disturbance, with respect to question A occurs. Finally, when a question about A is presented a second time, this anchor-adjustment process is applied again to produce the second answer to A.

Putting all of the steps together, we obtain the prediction equation

$$\begin{array}{ccc} p(A & = & x,B=y,A=z)={\left\|M_{z}\cdot U_{BA}^{†}\cdot M_{y}\cdot U_{BA}\cdot M_{x}\cdot U_{A}^{†}\cdot \psi_{0}\right\|}^{2},\\ & = & {\left\|P\left(A=z\right)P\left(B=y\right)P\left(A=x\right)\cdot \psi_{0}\right\|}^{2},\\ P\left(A=x\right) & = & U_{A}\cdot M_{x}U_{A}^{†}\\ P\left(B=y\right) & = & U_{B}\cdot M_{y}U_{B}^{†}\\ P\left(A=z\right) & = & U_{A}\cdot M_{z}U_{A}^{†}\\ U_{BA} & = & U_{B}^{†}\cdot U_{A}. \end{array}$$

## Generating predictions

The quantum model uses a Hamiltonian with two model parameters to form each unitary evaluation matrix (see S1 Appendix). The first of these determines a potential function that moves amplitudes in a systematic direction reflecting the validity of a statement; the second parameter determines the diffusion of amplitudes equally in both directions, which disperses the amplitudes from an initial state. The predictions, described in the next section, were generated as follows: We fixed the parameters for the unitary matrix *U*_*A*_ equal to the best fit values to the empirical data of the previous study; then we varied the parameter for the potential function for the unitary matrix *U*_*BA*_ across a sufficiently wide range to cover the AB squared correlations observed in the experiment.

The theoretical covariance between measurements of issues was computed using the theoretical expectation: *Cov*_*XY*_ = *E*[(*X* − *μ*_*X*_) ⋅ (*Y* − *μ*_*Y*_)] = ∑_*x*_*j*__∑_*y*_*j*__ *p*(*X* = *x*_*j*_, *Y* = *y*_*j*_) ⋅ (*x* − *μ*_*Y*_) (*y* − *μ*_*Y*_), where *μ*_*X*_ and *μ*_*Y*_ are the means of the two variables, and *p*(*X* = *x*_*j*_, *Y* = *y*_*j*_) was computed from the quantum model described in the previous section. The correlation was then computed by $r_{XY}=\frac{Cov(X,Y)}{Std(X)\cdot Std(Y)}$, where where *Std*(*X*) and *Std*(*Y*) are the standard deviations of the two variables. The variations in the potential function for B produced a wide range of squared correlations between A and B. This allowed us to examine the predicted relation between the squared correlation of A and B and the squared correlation between A and A. The general finding is that increasing the difference between the potential function parameters had the effect of systematically decreasing the squared correlation between the predicted ratings produced by the A and B statements as well as decreasing the squared correlation between the A and A ratings. This finding is robust and is not restricted to a fixed diffusion rate parameter. The same relation is obtained when we select different diffusion rate parameters.

**Fig 1:**
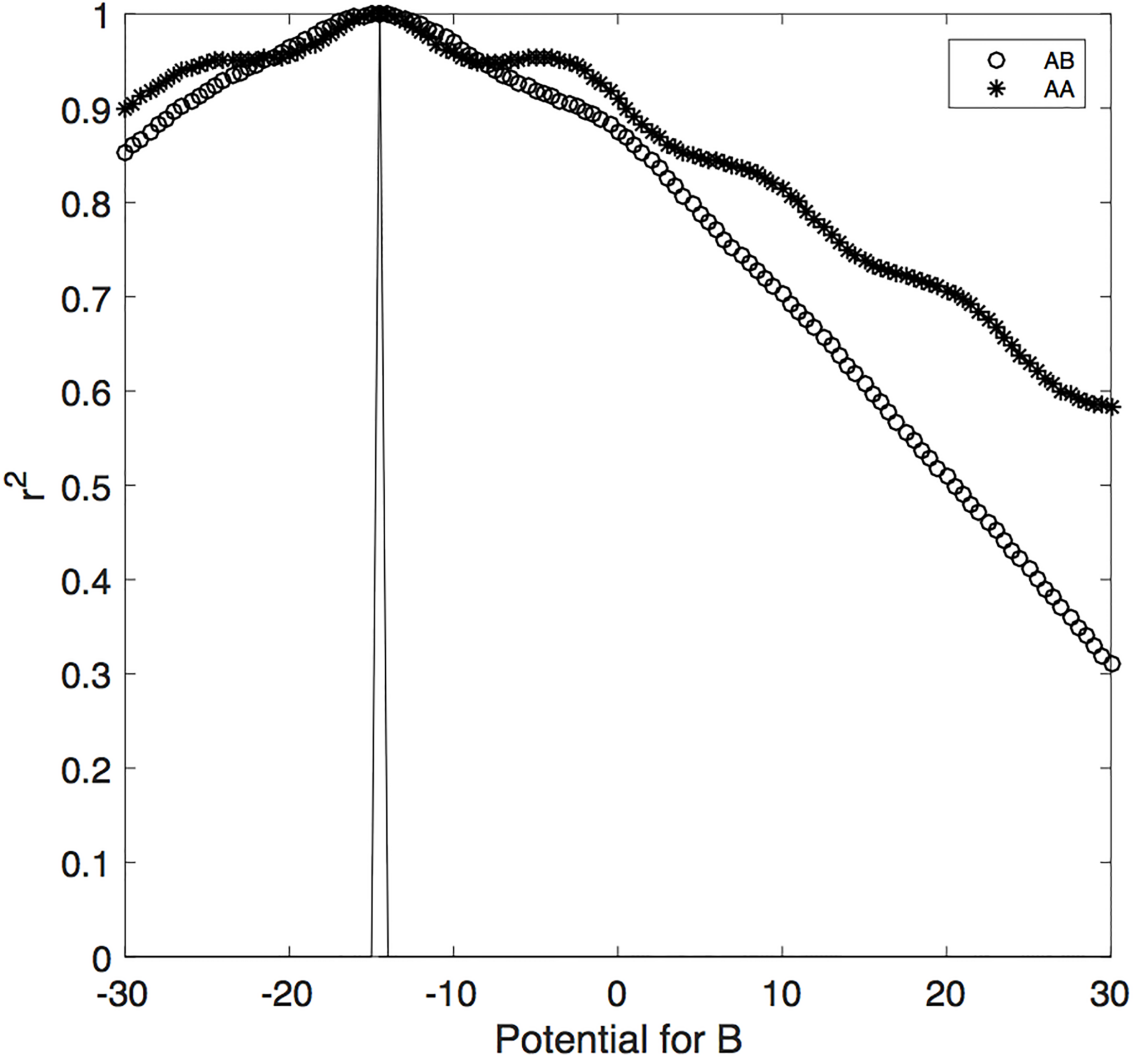
Predicted relation between AB and AA squared correlations. The horizontal axis represents the potential function value for B. The spike shows the location of the potential value for A. The vertical axis represents the squared correlation for AA (asterisks) and for AB (circles).

Fig 1 shows the systematic relation between the squared correlations and the difference in the potential function values. The spike indicates where the potentials for A and B are identical, and at this location the two variables are identical and compatible. Departures in the potential of B from this spike location produces monotonically decreasing correlations between A and B, as well as monotonically decreasing correlations between A and A. In sum, this previously established quantum model predicts that decreasing the squared correlation between A and B measurements also decreases the squared correlation between the first and the second A measurements.

How often can we expect the quantum model to produce a reversal of opinion? For a 9 point scale, a reversal would occur if the judgment for A on the first occasion changes from a rating (6, 7, 8, 9) above the midpoint (5) to a rating (1, 2, 3, 4) below the midpoint (or visa versa) on the second measurement of A. It turns out that the model does not predict that this happens very often. Even when the potentials for A and B are maximally different in Fig 1, so that the squared correlation between A and A equals.60, the probability of reversing opinion only equals.10. In other words, if we used a binary choice scale, where states (1, 2⋯, 50) are assigned to one choice *x* = 0, and the remaining states assigned to the other choice, then we would rarely expect to find inconsistent choices between the first and second measurements of A. So, according to the quantum model used here, changes in the direction (from agree to disagree or visa versa) of opinion to statement A can happen but they are uncommon in the ABA paradigm.

## Methods

### Participants

The participants were 325 master workers recruited from Amazon MTurk with a small amount of monetary compensation. To increase geographic representativeness, about 1/4 of the participants were recruited from each of the four regional divisions of the United States that is used by the United States Census Bureau (Northeast, Midwest, South, West). They were 18–72 years old (M = 34.25, SD = 12.56). Of those reported gender (99%), 53% percent reported to be female. The majority identified themselves as non-Hispanic White (69%), followed by non-Hispanic Black or African-American (8%), Asian or Pacific Islander (6%), Hispanic (4%), American Indian or Alaskan Native (1%). The rest did not provide racial information or identified themselves as “other.” This study was approved by the Institutional Review Board at the Ohio State University.

### Procedure

The experiment included 12 pairs of issues asking opinions about (1) presidential approval and country satisfaction, (2) support to affirmative action for women and for racial minorities, (3) hostility to white and black people, (4) happiness with life and with relationship, (5) abortion with and without a specified reason, (6) religious preferences of conservative Christians and liberals, (7) President Obama’s policies to improve economy and to reduce the budget deficit, (8) legal status of undocumented immigrants and assisting an undocumented immigrant, (9) learning from election debates and whether the debates are fun to watch, (10) A conflict between living in a modern society and being a devout Muslim or Christian, (11) perception of the democratic and the republican candidate in the presidential campaigning, and (12) using military force in Afghanistan and in Iraq. The exact questions asked are presented in S2 Appendix. Judgments were made on a 0 to 100 scale. These 12 pairs of issues were shown in previous research to produce order effects and thus are viewed as incompatible according to the quantum cognition view [7].

Each pair of issues, A and B, were presented using the ABA paradigm as mentioned earlier. Using the pair of presidential approval and country satisfaction as an example:

1. First, the computer screen provided a brief introduction before presenting the set of questions, “We’d like to know you opinions about Barack Obama and how things are going in this country today.
2. After the participant clicked the Next button, the next screen asked issue A for the first time, “Do you approve of the way Barack Obama is handling his job as president?” The participant answered the question using a slider on the screen where 0 = completely disapprove and 100 = completely approve. Below the question and the slider, there was a secondary task asking the participant to memorize a randomly generated three-digit number, such as 426.
3. The participant then clicked the Next button to move to the next page, where issue B was asked, “All in all, are you satisfied with the way things are going in this country today?” Again, a slider where 0 = complete dissatisfied and 100 = completely satisfied was provided to answer the question, followed by the secondary task of memorizing another randomly generated three-digit number, such as 519.
4. On the next page, the participant was asked to answer issue A for the second time along with responding to the secondary task, “Now given that you have just thought about how satisfied you are with the way things are going in this country today, what do you think AT THIS MOMENT, how much you approve of the way Barack Obama is handling his job as president? Please enter the previous two numbers first, then answer the question on the next page.” On this page, the participants typed down the two numbers they could remember.
5. Recall the two numbers that you were asked to remember.
6. Then on the final page, the issue A question was reiterated, “Now given that you have just thought about how satisfied you are with the way things are going in this country today, what do you think AT THIS MOMENT, how much you approve of the way Barack Obama is handling his job as president?” The same 0–100 slider anchored by “completely disapprove” and “completely approve.”

Each issue required less than a minute to complete. To discourage memorizing the judgments they were making, we included a number memory task to try to deplete the participants’ working memory when they were answering the questions. Participants were almost accurately recalled all the numbers that we asked them to memorize. Of course, this does not guarantee that they could not remember their judgments. In addition, the 0–100 point scales were used to try to make it more difficult to remember the previous judgment.

## Results

**Fig 2:**
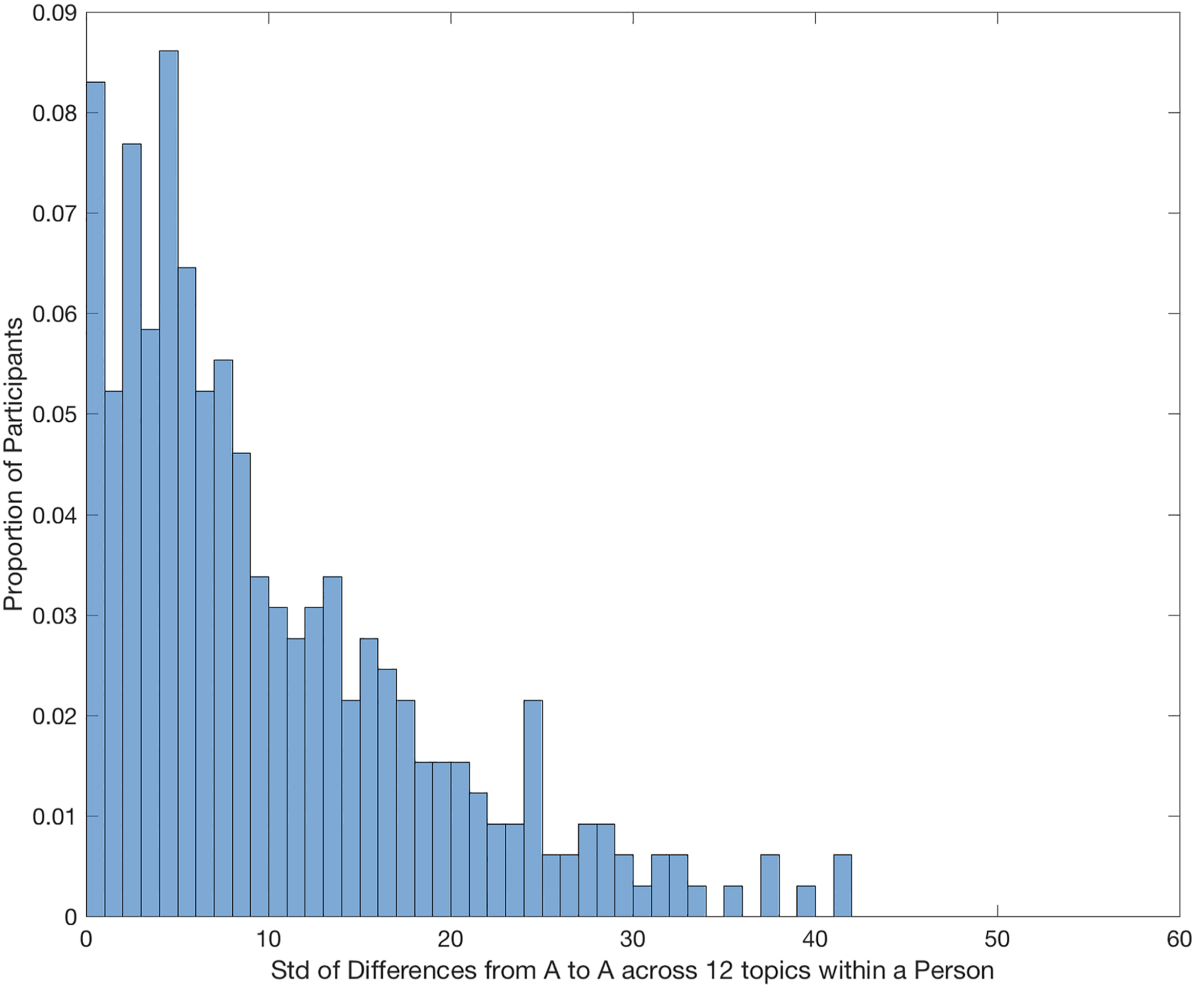
Standard deviation of change scores. The horizontal axis represents the standard deviation of the change score from the first to second measurement of issue A computed for each individual. The vertical axis represents the proportion across all participants of observing a value of the standard deviation.

First we examine the extent to which each individual changed his or her opinion across all 12 sets of issues. To do this, we computed the standard deviation of the change score *A*_1_ − *A*_2_ across all 12 sets of issues. Fig 2 presents the proportion of participants that produced each value of the standard deviation. As can be seen in the figure, there is a substantial amount of change occurring at the individual level. The mean is near 10 std on the 0 to 100 point scale. A quarter of the participants show changes less than 3.5 std, but a quarter also show changes larger than 15 std.

**Fig 3:**
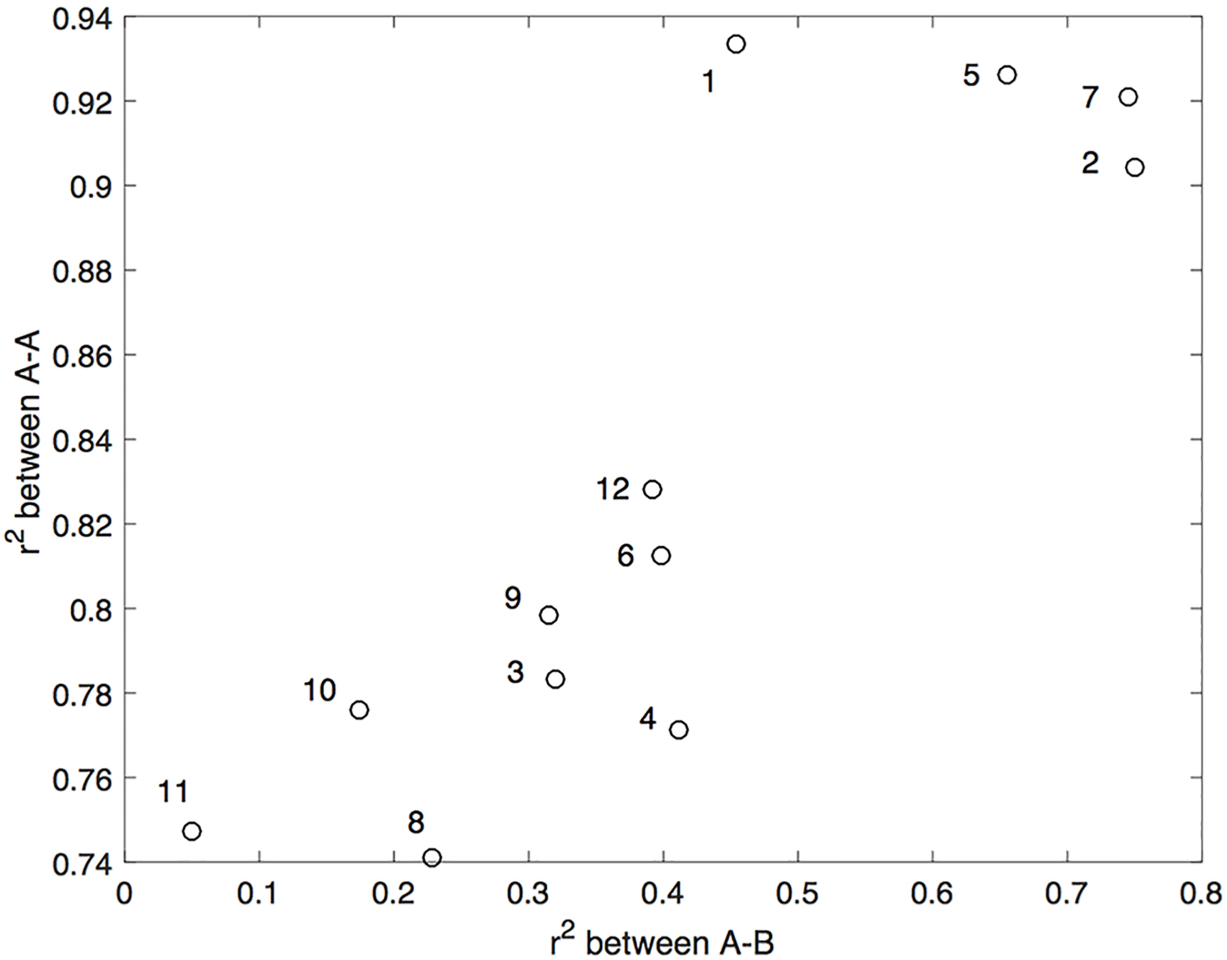
Relation between AB and AA correlations. The horizontal axis represents the 12 AB correlations, and the vertical axis represents the 12 AA correlations. The set of issues associated with each pair of correlations is indicate next to the corresponding point in the scatterplot.

Next for each of the 12 sets of issues, we examine the squared correlation between A and B (when A was measured the first time), and the squared correlation between the two measurements of A (with B measured in between). This produce 12 correlations between A and B, and 12 correlations between the two measurements of A. Each correlation was computed using the standard Pearson formula across 325 pairs obtained from the 325 participants. Fig 3 shows the relation between the 12 AB correlations and the 12 AA correlations. The number next to each point indicates the set of issues as described in the Method Section (e.g., 3 = hostility to white and black people). As can be seen in the figure, these two different types of correlations are strongly related. The correlation between the 12 pairs shown in Fig 3 equals.86 (*p* = .004). This relation generally agrees with the prediction of the quantum model. Note that the observed AA correlations are within the same range as the predicted correlations shown in Fig 1 when the potential for B is within the range −30 to 30.

Finally, we compare the correlations between AB (when A was measured the first time) versus between BA (when A was measured the second time). Although they are similar, in 10 out of the 12 cases, the BA correlation was higher than the AB correlations (*p* = .0386 according to a sign test). This order effect suggests that there was a tendency for the A ratings to become more similar to the B ratings after making the B ratings. This agrees with the anchor—adjustment property of the quantum model.

## Concluding comments

The purpose of this article was to empirically test a prediction derived from quantum probability theory as applied to human judgments for what is called the ABA experiment. The experiment involves initially taking an opinion measurement on issue A, followed by a measurement on another issue B, and finally the measurement of issue A is repeated. If issue B is incompatible with issue A, or in other words, if there are order effects when measuring A and B, then quantum probability theory predicts that the correlation between A on the first and second measurements should be less than one. Psychologically, this means the measurement of B disturbs the initial measurement of A, and re-introduces uncertainty with respect to A. This prediction was challenged by [4] who claimed that people would not change their mind, but their claim was not empirically tested by them. The current study provides the first empirical test of this quantum cognition model prediction.

The test of quantum theory was made more precise by deriving predictions from a previously established model for this task [5]. The previously established model predicts that the squared correlation between the first and second A measurements should be positively related to the squared correlation between the AB measurements.

The predictions were tested using 12 measurements of opinions on 12 different sets of issues that were shown in past research to produce order effects. The results for the ABA experiment presented above support the predictions of the quantum model [5]. First of all, most participants revealed a moderate amount of change in their opinions from the first measurement of A to the second measurement of A. Second, the squared correlations between the first and second measurements of A were found to be in the same range predicted by the previously established quantum model. Finally, the squared correlation between the first and second measurement of A was positively related to the squared correlation between the A and B measurements, as predicted by the previously established quantum model.

Classical test theory [8] also postulates variability in measurements across occasions. According to this view, the measurement instrument used to measure opinions—rating agreement with an issue on a 0 to 100 point scale—represents both a true opinion for a person that is fixed across repeated measurements plus some measurement error that randomly varies across repeated measurements. However, the measurement error model differs from the quantum model in a fundamental manner when considering the an AA condition (measure A twice without B in between). The projection principle of quantum probability now predicts a perfect correlation between the first and second measurements of A, whereas classical test theory allows measurement error to occur across repetitions and reduce the correlation below one. Classical test theory also fails to make any predictions about the nature of the intermediate measurement B. To produce uncertainty in the second measurement of A, quantum theory requires the B measurement to be incompatible with the A measurement. It is the combination of these highly testable predictions—perfect correlation for the AA condition, and less than perfect correlation for the ABA when A and B are incompatible (and A, B show order effects)—that is unique to quantum theory.

From a broader point of view, it is not surprising that people vary their judgments across the first and the second measurements of an issue when another issue is evaluated in between. Social psychological theories anticipated that context effects would occur in this type of situation [6]. The quantum model can be viewed as a formalization of these conceptual theories of contextualized cognition. The advantage of using a formal model is that it allows derivation of new and precise predictions that may not be intuitive and accessible to human minds and words, such as the new prediction between the AA correlations and the AB correlations tested here.

One final comment needs to added in response to an important issue raised by one of the reviewers. Apparently there is some misunderstanding concerning the empirical tests of order effects reported by [9]. These authors reported tests of reciprocity and double stochasticity implied by quantum models (see. [2] sections 2.1.2.4 and 2.2.5.4). However, the tests reported by [9] were limited to a very special case of quantum models—that is, models which assume that each projector representing a measurement outcome is represented by a single dimensional subspace (what they call non-degenerate observables). Models using one—dimensional projectors are considered “toy” models, which allow graphical illustrations. Actual quantum models of cognition generally assume that the projectors are multi-dimensional (see, e.g., [7]). In fact, the projectors for rating scales used in [5] and in this application are multi-dimensional. The results reported by [9] do not provide any empirical tests of actual quantum models that use multi-dimensional projectors.

## Supporting information

S1 Appendix Details about initial state and construction of unitary matrices used to generate predictions. (PDF)

S2 Appendix Detailed instructions and questions used in the experiment. All questions used 0–100 slider scales. See the Method for a detailed example. Also see the Method for the secondary task of memorizing numbers.
(PDF)
