# Supplementary material for: Is there a problem with quantum models of psychological measurements?
Source: PLoS One. 2017 Nov 8;12(11):e0187733. doi: 10.1371/journal.pone.0187733 (PMC5678685; doi:10.1371/journal.pone.0187733)
Supplement: S1 Appendix — (PDF) [file pone.0187733.s001.pdf]

S1 Appendix Details about initial state and construction of unitary matrices used to generate predictions.

The initial state at the beginning of a trial and before a question appears is defined as $\boldsymbol{\psi}(0) = [\psi_i(0)]$, with zeros assigned to all states except for $\frac{1}{\sqrt{11}}$ assigned to the 11 states $i = 45, ..., 50, ...55$ in the neighborhood of the neutral state corresponding to the middle rating $R = 5$.

The unitary matrices were constructed from a particular type of quantum random walk model called the Feynman crystal model. Unitary matrices can be constructed from a matrix exponential $\boldsymbol{U} = exp(-i \cdot \boldsymbol{H})$, where $\boldsymbol{H}$ is a Hamiltonian matrix (a Hermitian matrix). The Hamiltonian matrix we used, $\boldsymbol{H} = [h_{ij}]$, is a tri-diagonal matrix. The entries $h_{i-1,j} = \alpha$ above the diagonal and $h_{i+1,j} = \alpha$ below the diagonal allow diffusion of amplitudes to adjacent states. The entries on the diagonal $h_{ii} = \beta \cdot \left(\frac{i}{N}\right)$ serve as the potential function. The potential on the diagonal corresponds to a linear potential function that produces constant force in the direction determined by β. The off diagonal entries determine the diffusion rate. We used a Hamiltonian matrix $\boldsymbol{H}_A$ with parameters (α_A, β_A) for the one issue, and we used another Hamiltonian matrix $\boldsymbol{H}_B$ with parameters (α_B, β_B) for the other issue. In sum, this model entails only two parameters for each of the two different types of questions.

The best fitting parameters from [**?**] were used to generate the predictions. We set $\beta = -14.5725$, $\alpha = 4.5975$ for issue A, and we set $\alpha = 4.5975$ for issue B and varied β from -30 to $+30$ to sweep out the model predictions shown in Figure 1. The middle of the range for β on issue B corresponds to the value of β used for issue A, and so the questions become compatible at this point.
