# Supplementary material for: Is there a problem with quantum models of psychological measurements?
Source: PLoS One. 2017 Nov 8;12(11):e0187733. doi: 10.1371/journal.pone.0187733 (PMC5678685; doi:10.1371/journal.pone.0187733)
Supplement: S2 Appendix — All questions used 0–100 slider scales. See the Method for a detailed example. Also see the Method for the secondary task of memorizing numbers. (PDF) [file pone.0187733.s002.pdf]

**S2 Appendix Detailed instructions and questions used in the experiment. All questions used 0-100 slider scales. See the Method for a detailed example. Also see the Method for the secondary task of memorizing numbers.**

We would like to ask you some questions about your opinions. For each question, there is no right or wrong answer. It is all about your own opinions and feelings AT THE MOMENT. Your answers will only be examined in aggregation along with other several hundreds of people"s answers. For some of the questions, we will ask you twice. We try to understand how people may update what they think about an issue after considering another issue. You are free to change (or not to change) your answers to the question when it is asked the 2nd time. Remember, it is all about your opinions and feelings AT THE MOMENT when you answer a particular question.

1. Presidential approval and country satisfaction (A) Do you approve of the way Barack Obama is handling his job as president? (B) All in all, are you satisfied with the way things are going in this country today? (C) Now given that you have just thought about how satisfied you are with the way things are going in this country today, what do you think AT THIS MOMENT, how much you approve of the way Barack Obama is handling his job as president?

2. Support to affirmative action for women and for racial minorities: (A) Do you generally favor Affirmative Action programs for women? (B) Do you generally favor Affirmative Action programs for racial minorities? (C) Now given that you have just thought about Affirmative Action programs for racial minorities, what do you think AT THIS MOMENT how much you generally favor Affirmative Action programs for women?

3. Hostility to white and black people: (A) How many black people do you think dislike whites? (0 = only a few, 100 = almost all) (B) How many white people do you think dislike blacks? (0 = only a few, 100 = almost all) (C) Now given that you have just thought about how many white people dislike blacks, what do you think AT THIS MOMENT how many black people dislike whites?

4. Happiness with life and with relationship: (A) Are you happy with your life in general? (B) Are you happy with your romantic relationship? (C) Now given that you have just thought about how happy you are with your romantic relationship, what do you think AT THIS MOMENT how happy you are with your life in general?

5. Abortion with and without a specified reason: (A) Do you think it should be possible for a pregnant woman to obtain a legal abortion if she is married and does not want any more children? (B) Do you think it should be possible for a pregnant woman to obtain a legal abortion if there is a strong chance of serous defect in the baby? (C) Now given that you have just thought about whether it should be possible for a pregnant woman to obtain a legal abortion if there is strong chance of serious defect in the baby, what do you think AT THIS MOMENT whether it should be possible for a pregnant woman to obtain a legal abortion if she is married and does not want any more children?

6. Religious preferences of conservative Christians and liberals: (A) Conservative Christians have gone too far in trying to impose their religious values on the country. (B) Liberals have gone too far in trying to keep religion out of the schools and the government. (C) Now given that you have just thought about whether Liberals have gone too far in trying to keep religion out of the schools and the government, please rate your agreement level AT THIS MOMENT with the following statement: Conservative Christians have gone too far in trying to impose their religious values on the country.

7. President Obama"s policies to improve economy and to reduce the budget deficit:

(A) I am generally optimistic that Barack Obama”s policies will improve economic conditions in the country. (B) I am generally optimistic that Barack Obama”s policies will reduce the budget deficit over the long term. (C) Now given that you have just thought about whether you are generally optimistic that Barack Obama”s policies will reduce the budget deficit over the long term, please rate your agreement level AT THIS MOMENT with the following statement: I am generally optimistic that Barack Obama”s policies will improve economic conditions in the country.

8. Legal status of undocumented immigrants and assisting an undocumented immigrant: (A) We should allow undocumented immigrants who have been in the U.S. for several years to gain legal working status. (B) It a criminal offense for an American to assist an undocumented immigrant to remain in the U.S. (C) Now given that you have just thought about whether it should be a criminal offense for an American to assist an undocumented immigrant to remain in the U.S., please rate your agreement level AT THIS MOMENT with the following statement: We should allow undocumented immigrants who have been in the U.S. for several years to gain legal working status.

9. Learning from election debates and whether the debates are fun to watch: During election year: (A) The debates are helpful in learning about the candidates. (B) The debates are fun to watch. (C) Now given that you have just thought about whether the debates are fun to watch, please rate your agreement level AT THIS MOMENT with the following statement: The debates are helpful in learning about the candidates.

10. A conflict between living in a modern society and being a devout Muslim or Christian: (A) There is a natural conflict between being a devout Muslim and living in a modern society. (B) There is a natural conflict between being a devout Christian and living in a modern society. (C) Now given that you have just thought about whether there is a natural conflict between being a devout Christian and living in a modern society, please rate your agreement level AT THIS MOMENT with the following statement: There is a natural conflict between being a devout Muslim and living in a modern society.

11. Perception of the democratic and the republican candidate in the presidential campaigning: In the past presidential election campaign: (A) The democratic candidate was too personally critical of the republican candidate. (B) The republican candidate was too personally critical of the democratic candidate. (C) Now given that you have just thought about whether the republican candidate was too personally critical of the democratic candidate, please rate your agreement level AT THIS MOMENT with the following statement: The democratic candidate was too personally critical of the republican candidate.

12. Using military force in Afghanistan and in Iraq: (A) The U.S. made the right decision in using military force in Afghanistan. (B) The U.S. made the right decision in using military force in Iraq. (C) Now given that you have just thought about whether the U.S. made the right decision in using military force in Iraq, please rate your agreement level AT THIS MOMENT with the following statement: The U.S. made the right decision in using military force in Afghanistan.
